# An integrated multiomic approach as an excellent tool for the diagnosis of metabolic diseases: our first 3720 patients

**DOI:** 10.1038/s41431-022-01119-5

**Published:** 2022-05-25

**Authors:** Ligia S. Almeida, Catarina Pereira, Ruxandra Aanicai, Sabine Schröder, Tomasz Bochinski, Anett Kaune, Alice Urzi, Tania C. L. S. Spohr, Nikenza Viceconte, Sebastian Oppermann, Mohammed Alasel, Saeedeh Ebadat, Sana Iftikhar, Eresha Jasinge, Solaf M. Elsayed, Hoda Tomoum, Iman Marzouk, Anil B. Jalan, Agne Cerkauskaite, Rimante Cerkauskiene, Tinatin Tkemaladze, Anjum Muhammad Nadeem, Iman Gamal El Din Mahmoud, Fawzia Amer Mossad, Mona Kamel, Laila Abdel Selim, Huma Arshad Cheema, Omid Paknia, Claudia Cozma, Carlos Juaristi-Manrique, Pilar Guatibonza-Moreno, Tobias Böttcher, Florian Vogel, Jorge Pinto-Basto, Aida Bertoli-Avella, Peter Bauer

**Affiliations:** 1 CENTOGENE GmbH, 18055 Rostock, Germany; 2 Lady Ridgeway Hospital for Children, Colombo, Sri Lanka; 3 Medical Genetics Department, Faculty of Medicine, Ain Shams University, Cairo, Egypt; 4 Department of Pediatrics, Faculty of Medicine, Ain Shams University, Cairo, Egypt; 5 Alexandria University Children Hospital, Alexandria, Egypt; 6 Navi Mumbai Institute of Research In Mental And Neurological Handicap (NIRMAN) / Pediatric Geneticist, Navi Mumbai, India; 7 Faculty of Medicine, Vilnius University, Vilnius, Lithuania; 8 Department of Molecular and Medical Genetics, Tbilisi State Medical University, Tbilisi, Georgia; 9 Pediatric Gastroenterology, Hepatology and Nutrition, the Children’s Hospital and Institute of Child Health, Lahore, Pakistan; 10 Cairo University Children Hospital (Abu El Reesh Children’s Hospital), Metabolic, Neurology, Cairo, Egypt; 11 Department of Oncology, University Medical Center Rostock, Rostock, Germany

**Keywords:** Metabolic disorders, Next-generation sequencing

## Abstract

To present our experience using a multiomic approach, which integrates genetic and biochemical testing as a first-line diagnostic tool for patients with inherited metabolic disorders (IMDs). A cohort of 3720 patients from 62 countries was tested using a panel including 206 genes with single nucleotide and copy number variant (SNV/CNV) detection, followed by semi-automatic variant filtering and reflex biochemical testing (25 assays). In 1389 patients (37%), a genetic diagnosis was achieved. Within this cohort, the highest diagnostic yield was obtained for patients from Asia (57.5%, mainly from Pakistan). Overall, 701 pathogenic/likely pathogenic unique SNVs and 40 CNVs were identified. In 620 patients, the result of the biochemical tests guided variant classification and reporting. Top five diagnosed diseases were: Gaucher disease, Niemann-Pick disease type A/B, phenylketonuria, mucopolysaccharidosis type I, and Wilson disease. We show that integrated genetic and biochemical testing facilitated the decision on clinical relevance of the variants and led to a high diagnostic yield (37%), which is comparable to exome/genome sequencing. More importantly, up to 43% of these patients (*n* = 610) could benefit from medical treatments (e.g., enzyme replacement therapy). This multiomic approach constitutes a unique and highly effective tool for the genetic diagnosis of IMDs.

## Introduction

Inherited metabolic disorders (IMD) constitute a vast, complex, and important group of rare genetic diseases. The term “inborn error of metabolism” was first referenced as early as 1902 by Sir Archibald Garrod regarding four diseases: alkaptonuria, pentosuria, cystinuria, and albinism [1]. Approximately 60 years after Garrod’s premonitory dissertations, IMDs were rediscovered and slowly redefined as molecular diseases [2].

IMDs can be described as genetic disorders that cause disruption of a metabolic pathway presenting throughout a patient’s life span, from the prenatal period through adulthood [3]. IMDs can lead to disease either by accumulation of a toxic substrate proximal to the metabolic block, a deficiency of the product distal to the block, or a diversion of the substrate to an alternative pathway. Currently, over 1400 IMDs are recognized [4]. Although each disorder is individually rare, they represent a common group of diseases with a prevalence of 1 in 784–2555 [5–7].

Importantly, some IMDs are treatable [8, 9], and thus, many are included in newborn screening programs in various countries, which is pivotal for preventive health care [10]. The past decade has seen revolutionary changes in the discovery and diagnosis of IMDs, as well as in the development of new therapies [11]. The number of recognized IMDs and insights into their varying phenotypes are expanding rapidly using genomic, metabolomic, and deep phenotyping technologies, such as high-resolution and functional neuroimaging [12]. Early disease recognition and treatment are, in many cases, critical for preventing neurological impairment and/or death of the patient.

In order to diagnose IMDs comprehensively, we have implemented a next generation sequencing (NGS) panel with over 200 genes that integrates genetic and biochemical testing performed in the same laboratory. This approach allows the efficient diagnosis of more than 180 metabolic diseases. To our knowledge, there is no such integrated approach currently being offered in clinical practice.

Within this paper, we present our experience of the first 3720 patients in our clinical diagnostic setup. We recommend the use of this kind of multiomic approach as a first-line diagnostic tool for patients suspected of having IMDs.

## Materials and methods

### Patients

The current study has been conducted within a diagnostic setting. Additionally, in a follow-up step, de-identified data and samples were utilized. Accordingly, this did not require Institutional Review Board approval in our jurisdiction due to the nature in which the study was carried out. Written informed consent for genetic testing related to the disease(s) of the patient was obtained. Additionally, the consent form declaration included information regarding storage of the data and further data processing for research purposes. Written informed consent was given by patients, legal guardians, or referring physicians. The informed consent form is available in English and several other languages at https://www.centogene.com/downloads.html.

All consecutive index patients for whom this panel was performed and reported within the period of July 2019 until July 2021 were included in this study (*n* = 3720). Tested relatives were excluded from this analysis. Data regarding country of origin, family history, consanguinity, clinical phenotype, as well as panel testing results were extracted from our rare disease-centric Bio/Databank.

### Lab procedures

DNA was extracted from EDTA blood or from dried blood spots (DBS) on filter cards (CentoCard^®^) with QIASymphony using a magnetic bead-based method (Qiagen), with an acceptance criterion of minimum 3 ng/µl. Genomic DNA was enzymatically fragmented, and regions of interest were enriched using DNA capture probes (Twist Biosciences, custom design). The final indexed libraries were sequenced on an Illumina platform (MiSeq), with a sequencing quality parameter of 99.5% coverage of the targeted regions with a minimum read depth of 20x. The panel design included 206 genes (listed in the Supplementary Information). We included genes causing diseases in the different metabolic pathways, that are relatively frequent (also in our bio/databank), or treatable/potentially treatable disorders.

When deploying CentoMetabolic panel, the coding regions, 10 bp of flanking intronic sequences, and known coding and non-coding pathogenic/likely pathogenic (P/LP) variants based on ClinVar, Human Gene Mutation Database^®^ (HGMD), and CENTOGENE’s Bio/Databank [13], of the selected genes were targeted for analysis. Data analysis, including alignment to the hg19 human reference genome (Genome Reference Consortium GRCh37), variant calling, and annotation is performed using a validated in-house pipeline [14]. Variants with insufficient quality scores were confirmed via Sanger sequencing according to our established criteria [15].

A semi-automated filtering strategy for the SNV and small indels was used, with the variants fulfilling the following criteria being selected for further evaluation: (1) variants previously classified in CENTOGENE’s Bio/Databank as P/LP, or as variants of uncertain significance (VUS); (2) unclassified variants; (3) variants with minor allele frequency (MAF) < 1% including our healthy cohort, and in silico predictions of high/moderate impact on protein function; (4) all variants described as disease-causing by external databases (HGMD and ClinVar); (5) variants with adaptive boosting (ADA) and random forest (RF) scores from dbscSNV19 > 0.6, consistent with predictions of abnormal splicing; (6) exclusion of variants previously classified as (likely) benign. Our healthy cohort includes approximately 24,000 adult individuals (>18 years) who are said to be unaffected, had no linked human phenotype ontology (HPO) terms, and had genome/exome data available.

A similar strategy was applied for the CNV selection (<2% MAF): (i) homozygous deletions (0 copy number); (ii) heterozygous deletions (1 copy) and duplications (>2 copies), affecting >2 exons. The CNV detection algorithm has a sensitivity of above 95% for all homozygous deletions and heterozygous deletions/duplications spanning at least three consecutive exons, based on the internal validation dataset of 150 individuals. In the current cohort, CNVs were evaluated by visual inspection of the raw data in the integrative genome viewer (IGV). Usually, homozygous variants did not require confirmation by an orthogonal method. Heterozygous CNVs, were always confirmed by qPCR, multiplex ligation-dependent probe amplification (MLPA), or chromosomal microarray (CMA).

When a P/LP/VUS variant was detected with zygosity corresponding to the mode of inheritance of the related disorder, and a biochemical assay was available at CENTOGENE, determination of the corresponding enzymatic activity and/or biomarker concentration was performed to complement and assist on the variant classification. The enzymatic activities were determined either by fluorimetry or liquid chromatography coupled with mass spectrometry in dried blood spots (Supplementary Tables 1 and 2). The quantification of the biomarkers was performed in DBS using mass spectrometry. All tests were accredited according to ISO 15189 guidelines.

The selected variants were then evaluated with respect to their pathogenicity and causality and are classified into five classes (P, LP, VUS, likely benign, benign), according to the ACMG guidelines for variant classification [16]. For final variant classification and reporting, the phenotype of the patient (HPOs), clinical suspicion, and results from other lab tests (if provided) were taken into consideration.

Interpretation of the findings was performed in the clinical context, with reports being issued as: (i) positive, for patients with P/LP variant(s) explaining the phenotype(s); (ii) unclear, for cases with VUS compatible with the clinical phenotype (at least partially); (iii) negative, for cases with no relevant variant identified.

## Results

The implemented panel included 206 genes related to more than 180 metabolic diseases assembled in 18 metabolic categories, with predominance of the lysosomal disorders (48 genes), carbohydrate (35 genes), and amino acid/peptide metabolism disorders (33 genes) (Supplementary Table 3). A full list of the genes included in the panel is shown in the Supplementary Information.

With this panel, we tested a global cohort with patients that originated from 62 countries (Supplementary Table 4). Over 60% of the patients were referred from Africa and Asia (*n* = 1232, 33% and *n* = 1122, 30%, respectively). Other patients originated from Europe (*n* = 901, 24%), Latin America (*n* = 218, 6%), the Middle East (*n* = 201, 6%), and North America (*n* = 45, 1%) (Table 1).

**Table 1:** Demographics of the 3720 patients from this cohort.

Features	Cohort of all cases (*n* = 3720)
Age at onset	Range: Birth—34 years
0–5 years old	363 (9.7%)
6–16 years old	12 (0.3%)
Older than 16 years old	4 (0.1%)
Unknown	3341 (89.8%) g
Age at testing	
0–5 years old	2392 (64.3%)
6–16 years old	946 (25.4%)
Older than 16 years old	337 (9.1%)
Unknown	45 (1.2%)
Family history	
Positive	732 (19.6%)
Negative	135 (3.6%)
Unknown	2853 (76.7%)
Consanguinity	
Yes	1133 (30.4%)
No	724 (19.4%)
Unknown	1863 (50%)
Geographical origin	
North America	45 (1%)
Latin America	218 (6%)
Europe	901 (24%)
Middle East	201 (6%)
Asia	1122 (30%)
Africa	1232 (33%)
Australia	1 (0%)

We were particularly interested in age at onset, family history, and consanguinity, but in many cases, the information was not provided by the referring clinicians (Table 1). For example, in only half of the cases, data on consanguinity was available. In 30.4% (*n* = 1133), the presence of parental consanguinity was reported. Most of the patients from the cohort were children younger than 5 years (*n* = 2392; 64.3%). On average, symptoms started around 1 year of age (range 0 to 34 years), with an average age at testing of 3 years (range 1 month to 38 years).

**Fig. 1:**
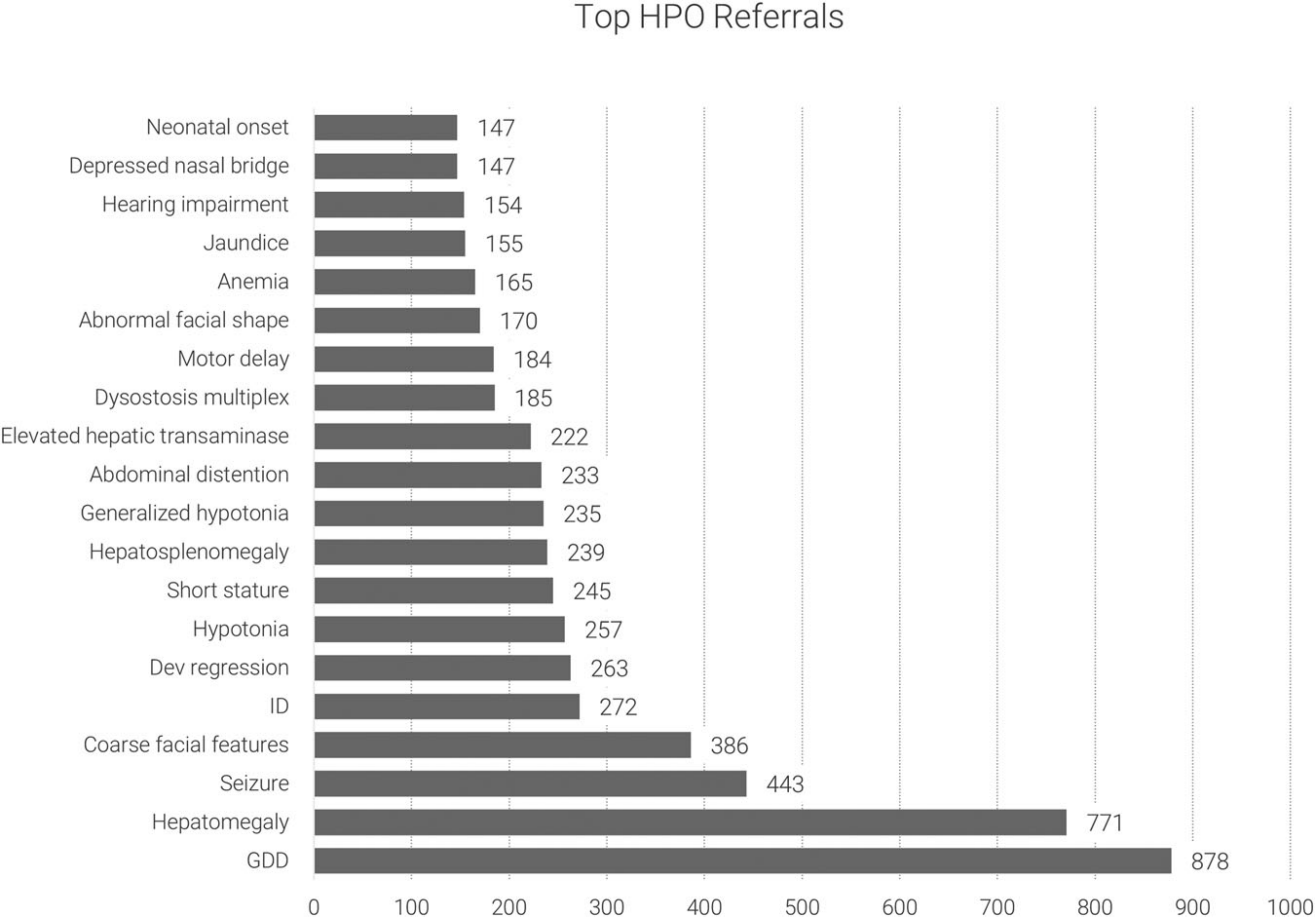
Top 20 referrals as provided in human phenotype ontology (HPO) terms. The most common HPO was global developmental delay (GDD), reported in 878 patients.

The main reasons for referrals are presented in Fig. 1 (HPOs). Neurological and non-neurological features were at the top of the list. Global developmental delay was the most frequently reported HPO (878 patients), followed by hepatomegaly (771 patients), seizures (443 patients), and coarse facial features (381 patients).

**Fig. 2:**
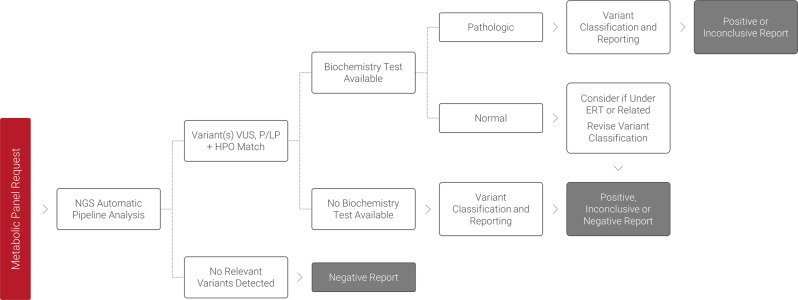
Semi-automated workflow used in the integrated multiomic approach. Flow chart illustrating the semi-automatic workflow integrating genetic and biochemical testing (enzyme replacement therapy—ERT).

After NGS of the genes in the panel, variant calling, and annotation, a semi-automatic filtering of the NGS data was performed considering variant frequency, type, classification, and zygosity, among others. Biochemical testing was then carried out if applicable (Fig. 2).

### Diagnostic yield, genetic variants, and biochemical testing

**Fig. 3:**
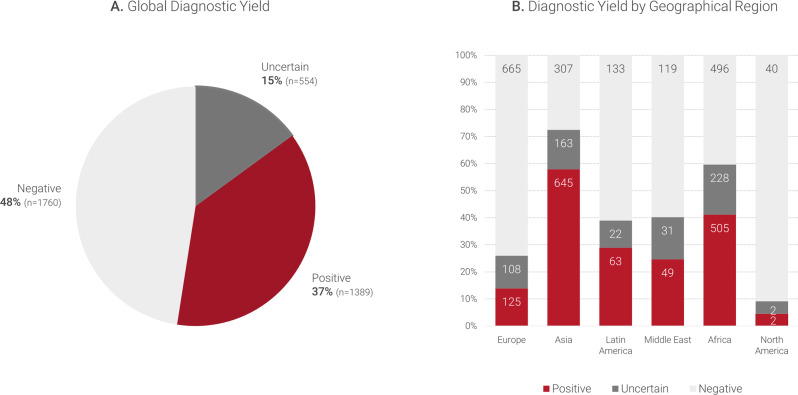
Diagnostic yield. **A** Diagnostic yield in the complete cohort. Overall, the diagnostic yield was 37% based on P/LP variants. **B** Diagnostic yield per geographic origin of the patients. The highest diagnostic yield was achieved in cases from Asia (mainly Pakistani patients), with nearly 60%.

A variant compatible with the phenotype was reported in 52% of the cases (*n* = 1943), from whom 37% (*n* = 1389) received a genetic diagnosis based on P/LP variants explaining the phenotype (Fig. 3A). Interestingly, the diagnostic yield varied per geographic origin, with the highest diagnostic yield obtained for patients from Asia (nearly 60%, *n* = 645), followed by patients from Africa (41%, *n* = 505), and from Latin America (29%, *n* = 63) (Fig. 3B).

We explored which factors could be contributing to the diagnostic yield by comparing features of the ‘positive’ and ‘negative’ cohorts. Besides differences observed in country of origin, positive family history and parental consanguinity, were more frequently reported in the positive group compared to the negative cohort (Supplementary Table 5).

**Fig. 4:**
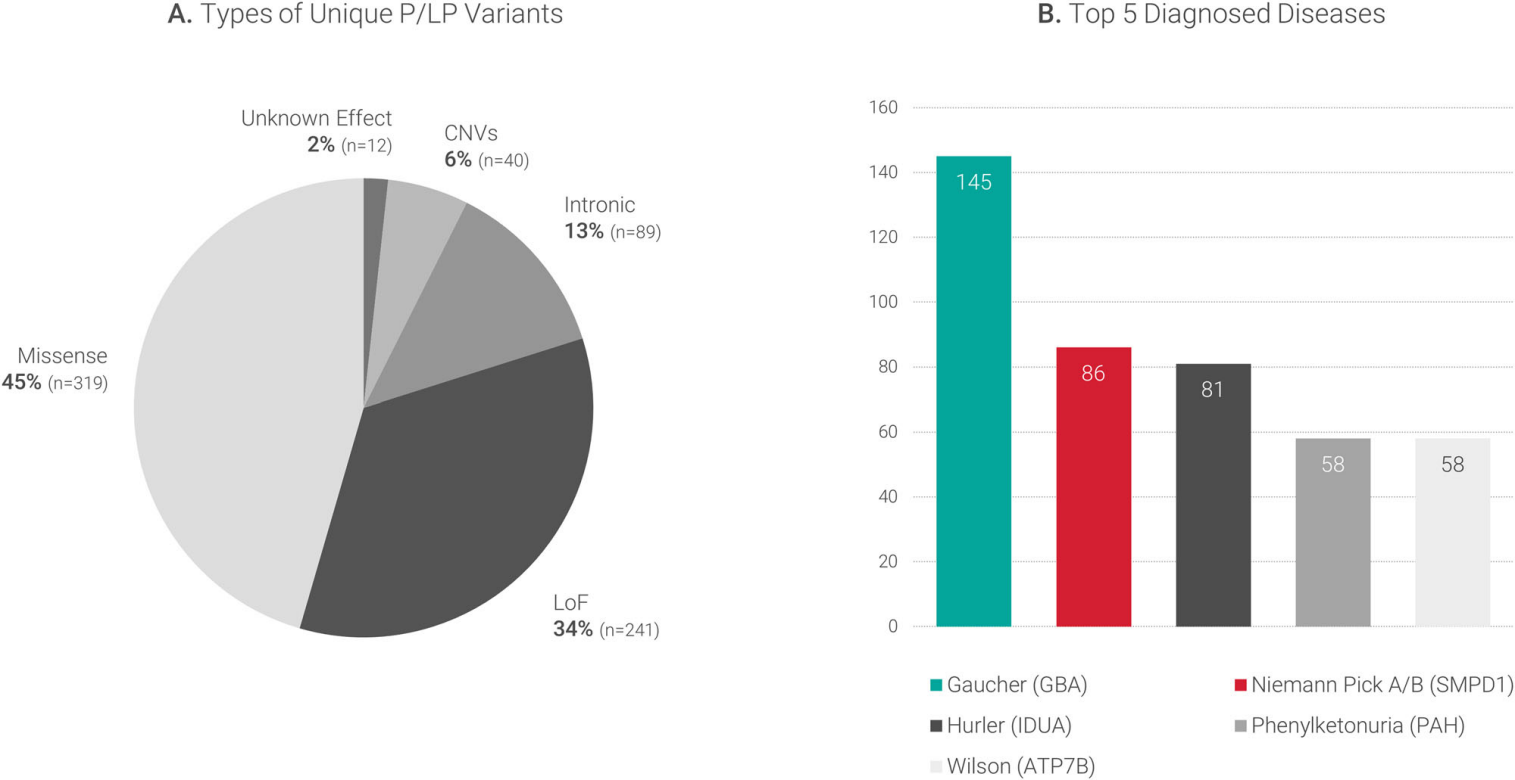
Variants identified in this study and main diagnosed diseases. **A** Unique P/LP variants identified in this study (loss of function (LoF), copy number variants (CNVs)). **B** Most frequent diseases diagnosed in this cohort.

We identified a total of 1415 P/LP variant occurrences, from which 701 were unique variants. Most of the P/LP variants were SNV (94%, *n* = 649) with missense, loss of function (LoF) variants (nonsense, frameshift, splicing), and other detected variants (Fig. 4A). In addition, we identified 40 unique CNVs (6%), given that the panel included detection of large CNVs based on the NGS data analysis. Most of the CNVs were deletions (85%, *n* = 34) and affected a single gene. However, in four cases, we found large CNVs affecting multiple genes and leading to unexpected diagnoses of distal trisomy 2p, trisomy 12p, Turner syndrome, and Xq microdeletion syndrome. These patients presented features such as hepatomegaly, hypoglycemia, hypertriglyceridemia (childhood or juvenile onset), nausea and vomiting, jaundice, macroglossia, and coarse facial features, which likely led to the referral of the patients with suspicion of a metabolic disorder. Recurrent CNVs were detected affecting the *IDS, IDUA, SLC3A1*, and *LDLR* genes (4x each), *GLB1* and *AGL* (3x each), *GALNS, ATP7A, CYP21A2*, and *SLC7A7* (2x each).

In some cases, the genetic diagnosis was based on the detection of a SNV and a CNV affecting the same gene. For example, in a 3-month-old male index with seizures, body odor, hyperisoleucinemia, hyperleucinemia, and hypervalinemia, we identified a heterozygous likely pathogenic frameshift variant and a heterozygous likely pathogenic deletion of exons 2-3 of the *DBT* gene. This, together with the specific clinical presentation strongly suggests the diagnosis of autosomal recessive maple syrup urine disease type II. Indeed, further carrier testing in the parents confirmed the trans phase of the alleles. In addition, a 2-year-old male index presenting with abdominal distention and splenomegaly had a heterozygous pathogenic missense variant and a heterozygous pathogenic deletion of exons 8-10 of the *GBA* gene. The activity of beta-glucocerebrosidase was extremely low, and the biomarker lyso-Gb1 was pathologically increased—establishing the diagnosis of Gaucher disease. The last patient of this group was a 1-year-old male that presented with global developmental delay, hepatosplenomegaly, cherry red spot of the macula, and hyperammonemia. We identified a pathogenic heterozygous deletion encompassing exons 1 to 17, and a pathogenic hemizygous frameshift variant, both affecting the *GLB1* gene. In addition, the activity of beta-galactosidase was pathologically reduced, confirming the genetic diagnosis of GM1-gangliosidosis type I.

### Usefulness of combined genetic/biochemistry testing

For over half of the 1415 variants classified as P/LP, biochemical testing was carried out (51.8%, *n* = 733). From the variants that were ultimately classified as P/LP, 176 were novel and located within genes with biochemical testing available. The results of the enzymatic and/or biomarker testing were essential to decide on the clinical relevance of the variant. For example, 23 and 20 novel variants were classified as P/LP in *SMPD1* and *NPC1*, respectively. The list of 176 novel variants is presented in Supplementary Table 6.

To further assess the impact of the biochemical testing, we evaluated all variants that had triggered a biochemical analysis, and the resulting variant classification and reports. In 620 patients, the diagnostic conclusion had been influenced by the result of the biochemical testing (Table 2). Having the biochemical result likely led to the low number of VUS reported in this cohort. Among those, in only 11 cases a VUS was reported, along with a negative or positive biochemical result. For example, we identified two heterozygous missense variants in *PSAP* in a 1-year-old patient with abdominal distention, hepatomegaly, and hyperlipidemia, and normal biomarker lyso-Gb1. The variants were reported (VUS) with recommendation to further parental targeted testing and biomarker lyso-Gb1 follow-up. Conversely, we also had cases with pathological enzymatic or biomarker testing, but the detected variants were classified as VUS based on the presence of contradictory evidence.

**Table 2:** Impact of combined genetic and biochemical testing on variant classification and reporting.

Combined genetic and biochemical testing	Reported positive (patients)	Reported negative (patients)	Reported inconclusive (patients)
Biochemisty with pathologic result	590	0	9
Biochemisty with normal result	0	19	2

A total of 590 patients that received a positive diagnosis (out of 1389, 42%), had a pathologic enzymatic/biomarker result that supported variant classification as P/LP.

In other cases, the result from biochemical testing was instrumental for excluding the clinical relevance of the variant(s). Specifically, in 19 patients, the identified variant was excluded from reporting after the enzymatic/biomarker determination was within normal values (Table 2). In some cases, intronic variants could be excluded (e.g. *GALNS*, NM_001323544.1:c.1020 + 307 G > C), and in others, the phase of two heterozygous variants was inferred as being in the same allele (cis configuration), as examples: *TPP1* (NM_000391.3:c.340 G > A, p.(Val114Met) and NM_000391.3:c.1033 A > C, p.(Met345Leu)).

More importantly, in 590 cases the pathological result of the biochemical tests guided the variant classification and final reporting. These 590 patients received a genetic diagnosis with supportive biochemical evidence. The most impacted gene was *GBA* (103 patients with Gaucher disease).

### Diseases detected in this cohort

Overall, 120 different diseases were diagnosed with the top five diseases being Gaucher disease, Niemann-Pick disease type A/B, mucopolysaccharidosis type I, phenylketonuria, and Wilson disease (Fig. 4B). Conversely, 30 diseases were ultra-rare in this cohort and were detected only once each. Most disorders were autosomal recessive (*n* = 102). All diseases that were diagnosed are provided in Supplementary Table 6.

Considering the different geographic regions, the top three diseases in patients from Asia and Africa were Gaucher disease, Niemann-Pick disease type A, Wilson disease (Asia), and phenylketonuria, Gaucher disease, and mucopolysaccharidosis type II (Africa). Surprisingly, phenylketonuria was one of the top three diseases in tested patients from Europe.

When combined, 300 patients were diagnosed with a type of mucopolysaccharidosis (*ARSB, GALNS, GLB1, GUSB, HGSNAT, IDS, IDUA, NAGLU*, and *SGSH* genes). Mucopolysaccharidosis type I (*IDUA* = 81 patients), type IVA (*GALNS* = 50 patients), and type II (*IDS* = 41 patients) were the most commonly diagnosed. Importantly, for five of these diseases there is enzyme replacement therapy (ERT) available. Thus, 200 of these patients would be eligible for treatment. Furthermore, from this cohort, 610 patients (43%, *n* = 1389) were diagnosed with diseases which are treatable.

We also investigated the time between the onset of symptoms and the establishment of a genetic diagnosis (often referred to as a “diagnostic odyssey”). Data was available for 126 patients with positive reports. On average, these patients waited 2 years from the described onset of symptoms until a diagnosis could be established, with a range from 0 to 20 years. Five patients waited over 10 years to receive a diagnosis. These cases were from Asia, Africa, and Latin America with Dubin-Johnson syndrome, alkaptonuria, fucosidosis, Gaucher disease, and phenylketonuria.

## Discussion

IMDs can present at any time, from the antenatal period to adulthood, with insidious or acute presentations, and with a wide clinical spectrum ranging from unspecific neurodevelopmental delay to acute metabolic decompensation and death. Early disease recognition, accurate diagnosis, and treatment are lifesaving in many cases [17].

The use of NGS panels for phenotypically related disorders can increase the likelihood of identifying an underlying genetic cause and may be preferred to exome or genome sequencing to maximize target coverage and avoid secondary findings [18]. Traditionally, the diagnosis of metabolic diseases is achieved via genetic testing (NGS panels—Sanger sequencing) or biochemical testing, in patients with a clinical presentation suggestive of an IMD. Current testing methods for IMDs comprise a stepwise strategy starting with molecular and followed by biochemical testing or vice versa [19] (ACMG Statements and Guidelines—https://www.nature.com/collections/afebagafci).

Approaches that combine genetic and metabolomic/biochemical data have been suggested as highly beneficial for the diagnosis of IMDs [17, 20, 21], but to our knowledge, this multiomic approach has not been implemented in the clinical practice so far.

In this study, we describe our applied tool with integrated genetic and biochemical testing for the diagnosis of genetic metabolic disorders. This approach has several advantages: (i) convenient use of filter cards (CentoCard) for handling, transportation, and genetic—biochemical testing using DBS; (ii) simultaneous detection of SNVs and CNVs; (iii) semi-automatic filtering of variants, with subsequent biochemical workflow activation in the same laboratory; (iv) comprehensive interpretation and classification of genetic variants; and (v) shortened diagnostic odyssey by avoiding stepwise testing.

Within this study, we report the results of our first 3720 patients. The obtained diagnostic yield of 37%, with genetic diagnoses established in 1389 patients, is comparable or even higher than the average reported yield after exome sequencing [14, 22]. Within this cohort, the diagnostic yield raised to nearly 60% in patients that originated from Asia (mainly Pakistan), which is also comparable to the results from exome sequencing in patients from the same area and hospital [23]. This could be due to the specific referral centers some of them with specialization on metabolic disorders, and the simultaneous lack of local resources for neonatal screening and genetic testing. Other factors such as the presence of parental consanguinity and positive family history are influencing the diagnostic yield (Supplementary Table 5), as described by us before [14, 23].

We identified 1415 P/LP variant occurrences; for 51.8% of them, a biochemical test was performed. In the majority of the patients, the variant classification as P/LP was supported by the result of the enzymatic/biomarker testing (Table 2). This was particularly relevant for intronic variants and novel missense variants, which are usually classified as VUS, in the absence of additional evidence (Table 2). In addition, the biochemical testing allowed the exclusion of other genetic variants based on normal test results.

Our results also contribute to increased knowledge on the genetic etiology of rare diseases. Taking peroxisomal biogenesis disorders as an example, the Zellweger spectrum disorder is caused by pathogenic variants in 13 known ZSD-PEX genes [24]. According to the literature *PEX1* is responsible for over 60% of the cases. However, in our cohort *PEX12* is the major gene involved, with five patients diagnosed (Supplementary Table 6). The wide spectrum of novel causative genetic variants reported here is likely due to the testing of a global cohort, with inclusion of patients from geographic regions that usually have limited access to molecular testing resources and are underrepresented in the scientific literature and genetic databases.

We also confirm previous findings on specific established pathogenic variants. For example, Gaucher disease was the most frequently detected disorder in our cohort, with 126 newly diagnosed patients. Over half of them were homozygote or compound heterozygotes for a single specific *GBA* pathogenic variant (NM_000157.3:c.1448 T > C, p.(Leu483Pro)), which is known to be among the four most common pathogenic variants in *GBA* [25]. Of note, we also detected seven novel P/LP variants in *GBA*.

Unexpectedly, phenylketonuria was among the top five diseases detected in this cohort (58 patients, Fig. 4B), and it was diagnosed mainly in tested patients from Africa (Egypt). In addition, this is one of the top three diseases being diagnosed in tested patients from Europe (Georgia and Romania). Phenylketonuria is an excellent example of a treatable disease and a model for early detection and treatment, as this was one of the first diseases covered by newborn screening programs in the early 1960s [26, 27].

The aim for newborn screening is early detection of treatable disorders, and a small group of metabolic disorders is usually included in the different screening programs. This ensures that at least part of the infants with IMDs are diagnosed before the onset of symptoms—significantly improving the options for therapy and health outcomes [28]. Unfortunately, newborn screening is not well-established in most resource limited countries. The fact that phenylketonuria is still a common disorder being diagnosed in our study cohort illustrates the challenges that mainly developing countries have with implementation and expansion of their newborn screening programs (major problems are financial issues, medical and logistical support, society education, policy development, program evaluation, and sustainability) [27].

As such, our integrated diagnostic approach is relevant for improvement of the diagnosis and early detection of IMDs. This is even more relevant for groups of IMDs, such as mucopolysaccharidosis, aminoacidopathies, and organic acidemias, amongst others, that are treatable via nutritional therapy, vitamin and trace element substitution, enzyme replacement therapy, hematopoietic stem cell transplant, solid organ transplantation, pharmacological therapy, and gene-based therapy, among others [8]. This holds especially true as one of the most effective treatments are nutritional, which are relatively affordable, widely available, and in many cases, highly effective [8]. It is striking that four of the top five diseases have specific treatments, such as dietary management, enzyme replacement therapy, and substrate reduction therapy, among others.

In conclusion, we show that combined genetic and biochemical testing facilitated the decision on clinical relevance of the variants and led to a high diagnostic yield (37%). The established genetic diagnosis in nearly 1400 patients is expected to lead to benefits in treatment and/or counseling for the families. Future approaches should integrate transcriptomics, proteomics, and/or metabolomics for an enhanced detection and treatment of IMDs.

## Supplementary information

Supplemental material

## Data Availability

The de-identified data set is available on request to the corresponding author.
