# Supplementary material for: An integrated multiomic approach as an excellent tool for the diagnosis of metabolic diseases: our first 3720 patients
Source: Eur J Hum Genet. 2022 May 25;30(9):1029–35. doi: 10.1038/s41431-022-01119-5 (PMC9437014; doi:10.1038/s41431-022-01119-5)
Supplement: Supplementary file 1 — Supplemental material [file 41431_2022_1119_MOESM1_ESM.docx]

**Supplementary Information**

**List of genes included in the CentoMetabolic® panel**

*ABCA1, ABCB4, ABCC2, ABCD1, ABCD4, ABCG5, ABCG8, ACAT1, ADA, AGA, AGL, AGPS, AGXT, ALAD, ALAS2, ALDH4A1, ALDOA, ALDOB, ALG3, ALPL, ANTXR2, APOA2, APOA5, APOB, APOC2, APOE, ARG1, ARSA, ARSB, ASAH1, ASL, ASS1, ATP7A, ATP7B, BCKDHA, BCKDHB, BTD, CBS, CD320, CETP, CLN3, CLN5, CLN6, CLN8, CPOX, CPS1, CPT1A, CTNS, CTSA, CTSD, CTSK, CYP11B1, CYP17A1, CYP19A1, CYP21A2, DBT, DDC, DHCR7, DIABLO, DLX4, DNAJC5, DPYD, ENO3, ENPP1, EPHX2, ETHE1, FAH, FBP1, FECH, FGF23, FUCA1, G6PC, G6PD, GAA, GALC, GALE, GALK1, GALNS, GALT, GAMT, GATM, GBA, GBE1, GHR, GK, GLA, GLB1, GM2A, GNPAT, GNPTAB, GNPTG, GNS, GUSB, GYG1, GYS1, GYS2, HCFC1, HEXA, HEXB, HFE, HJV, HGD, HGSNAT, HLCS, HMBS, HPD, HPRT1, HSD3B2, HYAL1, IDS, IDUA, ITIH4, IVD, KHK, LAMP2, LCAT, LDHA, LDLR, LDLRAP1, LIPA, LIPC, LIPI, LMBRD1, LPA, LPL, MAN2B1, MANBA, MCOLN1, MFSD8, MMAA, MMAB, MMACHC, MMADHC, MMUT, NAGA, NAGLU, NAGS, NEU1, NPC1, NPC2, OTC, PAH, PCSK9, PDHB, PEX1, PEX10, PEX12, PEX13, PEX14, PEX16, PEX19, PEX2, PEX26, PEX3, PEX5, PEX6, PEX7, PFKM, PGAM2, PGK1, PGM1, PHKA1, PHKA2, PHKB, PHKG2, PKLR, PNPO, POR, PPOX, PPP1R17, PPT1, PRKAG2, PSAP, PYGL, PYGM, RBCK1, SGSH, SI, SLC17A5, SLC22A5, SLC25A13, SLC25A15, SLC25A20, SLC25A36, SLC2A1, SLC2A2, SLC2A3, SLC37A4, SLC3A1, SLC3A2, SLC40A1, SLC6A19, SLC6A8, SLC7A7, SLC7A9, SLCO1B1, SLCO1B3, SMPD1, SUMF1, TAT, TFR2, TPP1, UGT1A1, UMPS, UROD, UROS*

**Supplementary Table 1**. Enzymatic assays included in the panel.

| Enzyme Assay | Disease | Product Detection method | Gene |
| --- | --- | --- | --- |
| N-acetyl-alpha-glucosaminidase | MPS IIIB | Fluorimetry | *NAGLU* |
| Palmitoyl-protein thioesterase | Neuronal ceroid lipofuscinosis type 1 | Fluorimetry | *PPT1* |
| Tripeptidyl peptidase | Neuronal ceroid lipofuscinosis type2 | Fluorimetry | *TPP1* |
| Acid lipase | Wolman | Tandem –MS | *LAL/LIPA* |
| Acidic sphingomyelinase | Niemann-Pick Type A/B | Tandem-MS | *SMPD1* |
| Acidic α-glucosidase | Pompe | Fluorimetry | *GAA* |
| Alpha-fucosidase | Alpha-fucosidase deficiency | Fluorimetry | *FUCA1* |
| Alpha-mannosidase | Alpha-mannosidase deficiency | Fluorimetry | *MAN2B1* |
| alpha-N-acetylgalactosaminidase | Schindler/Kanzaki | Fluorimetry | *NAGA* |
| Arylsulfatase B | MPS VI | Fluorimetry | *ARSB* |
| Beta-galactosidase | MPS IV B | Tandem-MS | *GLB1* |
| Beta-glucuronidase | MPS VII | Fluorimetry | *GUSB* |
| Beta-hexosaminidase | Tay-Sachs | Fluorimetry | *HEXA* |
| Beta-mannosidase | Beta-mannosidase deficiency | Fluorimetry | *MANBA* |
| Galactocerebrosidase | Krabbe | Tandem-MS | *GALC* |
| Iduronate-2-sulfatase | MPS II | Fluorimetry | *IDS* |
| N-acetylgalatosamine-6-sulfate-sulfatase | MPS IVA | Tandem-MS | *GALNS* |
| Total hexaminidase | Sandhoff | Fluorimetry | *HEXB* |
| α-galactosidase | Fabry | Fluorimetry | *GLA* |
| α-L-Iduronidase | MPS I | Fluorimetry | *IDUA* |
| β-glucocerebrosidase | Gaucher | Fluorimetry | *GBA* |

**Supplementary Table 2.** Biomarker determinations included in the panel.

| Biomarker determination | Disease | Detection method | Genes |
| --- | --- | --- | --- |
| Glucosylsphingosine (lyso-Gb1) | Gaucher | Tandem-MS | *GBA* |
| Lyso-Ceramide trihexoside (lyso-Gb3) | Fabry | Tandem-MS | *GLA* |
| Lyso-SM-509 | Niemann-Pick | Tandem-MS | *SMPD1, NPC1, NPC2* |
| 3-O-methyldopa (3-OMD) | AADC deficiency | Tandem-MS | *DDC* |

**Supplementary Table 3.** Metabolic categories, following SSIEM classification of IMDs, covered by 206 genes included in the panel.

| Type of metabolic disorders covered | Number of genes |
| --- | --- |
| Lysosomal disorders | 48 |
| Disorders of carbohydrate metabolism | 35 |
| Disorders of amino acid and peptide metabolism | 33 |
| Disorders of the metabolism of sterols | 16 |
| Peroxisomal disorders | 16 |
| Disorders in the metabolism of vitamins and (non-protein) cofactors | 10 |
| Disorders of lipid and lipoprotein metabolism | 8 |
| Disorders of porphyrin and haem metabolism | 8 |
| Defects in Hormone Biogenesis or Function | 7 |
| Disorders in the metabolism of purines, pyrimidines and nucleotides | 6 |
| Disorders in the metabolism of trace elements and metals | 6 |
| Disorders of energy metabolism | 6 |
| Disorder of phosphate, calcium and vitamin D metabolism | 3 |
| Disorders of fatty acid and ketone body metabolism | 3 |
| Congenital disorders of glycosylation and other disorders of protein modification | 2 |
| Defects in cholesterol and lipoprotein metabolism | 2 |
| Disorders of neurotransmitter metabolism | 1 |
| Porphyria and Bilirubinemia | 1 |

**Supplementary Table 4**. List of 62 countries and geographic regions from the patients included in this study.

| EUROPE | ASIA | AFRICA | MIDDLE EAST | LATIN AMERICA | NORTH AMERICA | AUSTRALIA |
| --- | --- | --- | --- | --- | --- | --- |
| Albania | India | Algeria | Iran | Argentina | Canada | Australia |
| Azerbaijan | Indonesia | Egypt | Irak | Bolivia | USA |  |
| Bulgaria | Kazakhstan | Kenya | Israel | Brazil |  |  |
| Germany | Pakistan | Libya | Jordan | Colombia |  |  |
| Georgia | Philippines | Morocco | Kuwait | Costa Rica |  |  |
| Greece | Korea | Rwanda | Lebanon | Ecuador |  |  |
| Hungary | Sri Lanka | Sudan | Oman | Mexico |  |  |
| Ireland |  | Tanzania | Syria | Panama |  |  |
| Italy |  | Tunisia | UAE | Paraguay |  |  |
| Latvia |  | South Africa | Saudi Arabia | Peru |  |  |
| Lithuania |  |  |  | Venezuela |  |  |
| Malta |  |  |  |  |  |  |
| Rep. of Moldova |  |  |  |  |  |  |
| Poland |  |  |  |  |  |  |
| Romania |  |  |  |  |  |  |
| Spain |  |  |  |  |  |  |
| Slovenia |  |  |  |  |  |  |
| Switzerland |  |  |  |  |  |  |
| Turkey |  |  |  |  |  |  |
| Ukraine |  |  |  |  |  |  |
| United Kingdom |  |  |  |  |  |  |
|  |  |  |  |  |  |  |
| Total 62 countries | |  |  |  |  |  |

Supplementary Table 5. Demographics of the positive and negative cohorts.

| Features | Positive Cohort  (n=1389) | Negative Cohort  (n=1760) |
| --- | --- | --- |
| **Age at testing** | Range: Birth- 81 years | Range: Birth– 69 years |
| *0-5 years old* | 65% (910) | 64% (1132) |
| *6-16 years old* | 27% (370) | 24% (418) |
| *Older than 16 years old* | 7% (93) | 10% (176) |
| *Unknown* | 1% (16) | 2% (34) |
| **Family history** |  |  |
| *Positive* | 22% (302) | 16% (282) |
| *Negative* | 2% (27) | 5% (87) |
| *Unknown* | 76% (1060) | 79% (1391) |
| **Consanguinity** |  |  |
| *Yes* | 43% (598) | 19% (341) |
| *No* | 14% (197) | 24% (415) |
| *Un7known* | 43% (594) | 57% (1004) |
| **Geographical origin** |  |  |
| *North America* | 1% (2) | 2% (40) |
| *Latin America* | 5% (63) | 8% (133) |
| *Europe* | 9% (125) | 37% (665) |
| *Middle East* | 3% (49) | 8% (119) |
| *Asia* | 46% (645) | 17% (307) |
| *Africa* | 36% (505) | 28% (496) |

**Supplementary Table 6.** P/LP novel variants in genes with biochemical evidence for pathogenicity.

| Gene | Ref.Seq. and Nucleotide Change | Protein Change | Biochemical Abnormalities | No. of observations | Disease | MOI | Class |
| --- | --- | --- | --- | --- | --- | --- | --- |
| *ARSB* | NM_000046.3: c.1447G>A | p.(Glu483Lys) | enz. | 1 | Mucopolysaccharidosis type VI | AR | P |
| *ARSB* | NM_000046.3: c.166G>A | p.(Gly56Ser) | enz | 2 | Mucopolysaccharidosis type VI | AR | P |
| *ARSB* | NM_000046.3: c.278C>A | p.(Pro93Gln) | enz | 1 | Mucopolysaccharidosis type VI | AR | LP |
| *ARSB* | NM_000046.3: c.511_512del | p.(Gly171fs*) | enz | 1 | Mucopolysaccharidosis type VI | AR | P |
| *ARSB* | NM_000046.3: c.705C>G | p.(Tyr235*) | enz | 1 | Mucopolysaccharidosis type VI | AR | P |
| *ARSB* | NM_000046.3: c.980G>A | p.(Arg327Gln) | enz | 1 | Mucopolysaccharidosis type VI | AR | P |
| *ARSB* | NM_000046.4: c.1211del | p.(Pro404Argfs*141) | enz | 1 | Mucopolysaccharidosis type VI | AR | LP |
| *ARSB* | NM_000046.4: c.158A>G | p.(Asp53Gly) | enz | 1 | Mucopolysaccharidosis type VI | AR | P |
| *ARSB* | NM_000046.4: c.269_283dup | p.(Leu90_Ser94dup) | enz | 1 | Mucopolysaccharidosis type VI | AR | LP |
| *FUCA1* | NM_000147.4: c.1100del | p.(Glu367Glyfs*42) | enz | 1 | Fucosidosis | AR | P |
| *GAA* | NM_000152.3: c.119G>A | p.(Arg40Gln) | enz | 1 | Glycogen storage disease II | AR | P |
| *GAA* | NM_000152.3: c.1724A>T | p.(Tyr575Phe) | enz | 1 | Glycogen storage disease II | AR | P |
| *GAA* | NM_000152.3: c.1870C>T | p.(Leu624Phe) | enz | 1 | Glycogen storage disease II | AR | P |
| *GAA* | NM_000152.3: c.2646+1G>A |  | enz | 1 | Glycogen storage disease II | AR | P |
| *GAA* | NM_000152.3: c.695T>G | p.(Leu232Arg) | enz | 1 | Glycogen storage disease II | AR | LP |
| *GAA* | NM_000152.3: c.837G>A | p.(Trp279*) | enz | 1 | Glycogen storage disease II | AR | P |
| *GALC* | NM_000153.3: c.1021del | p.(Val341Serfs*18) | enz | 1 | Krabbe disease | AR | P |
| *GALC* | NM_000153.3: c.1270C>T | p.(Gln424*) | enz | 1 | Krabbe disease | AR | P |
| *GALC* | NM_000153.3: c.1274T>A | p.(Val425Glu) | enz | 1 | Krabbe disease | AR | LP |
| *GALC* | NM_000153.3: c.1835-2A>G |  | enz | 1 | Krabbe disease | AR | P |
| *GALC* | NM_000153.3: c.442G>A | p.(Gly148Arg) | enz | 1 | Krabbe disease | AR | LP |
| *GALC* | NM_000153.3: c.468G>T | p.(Trp156Cys) | enz | 1 | Krabbe disease | AR | LP |
| *GALC* | NM_000153.3: c.593A>C | p.(Glu198Ala) | enz | 1 | Krabbe disease | AR | P |
| *GALC* | NM_000153.3: c.758A>G | p.(His253Arg) | enz | 1 | Krabbe disease | AR | P |
| *GALC* | NM_000153.3: c.968G>A | p.(Gly323Glu) | enz | 1 | Krabbe disease | AR | LP |
| *GALNS* | NM_001323544.1: c.1275_1285delinsTG | p.(Pro426_Asn429delinsAsp) | enz | 1 | Mucopolysaccharidosis type IVA | AR | P |
| *GALNS* | NM_001323544.1: c.1357G>A | p.(Asp453Asn) | enz | 1 | Mucopolysaccharidosis type IVA | AR | P |
| *GALNS* | NM_001323544.1: c.1383-2A>G |  | enz | 1 | Mucopolysaccharidosis type IVA | AR | P |
| *GALNS* | NM_001323544.1: c.262+1G>T |  | enz | 1 | Mucopolysaccharidosis type IVA | AR | LP |
| *GALNS* | NM_001323544.1: c.462_467del | p.(Gln154_Phe155del) | enz | 1 | Mucopolysaccharidosis type IVA | AR | P |
| *GALNS* | NM_001323544.1: c.464T>G | p.(Phe155Cys) | enz | 1 | Mucopolysaccharidosis type IVA | AR | P |
| *GALNS* | NM_001323544.1: c.490G>T | p.(Glu164*) | enz | 4 | Mucopolysaccharidosis type IVA | AR | P |
| *GALNS* | NM_001323544.1: c.506C>T | p.(Pro169Leu) | enz | 1 | Mucopolysaccharidosis type IVA | AR | P |
| *GALNS* | NM_001323544.1: c.772G>A | p.(Gly258Arg) | enz | 1 | Mucopolysaccharidosis type IVA | AR | P |
| *GALNS* | NM_001323544.1: c.909del | p.(Glu304Asnfs*21) | enz | 1 | Mucopolysaccharidosis type IVA | AR | P |
| *GALNS* | NM_001323544.1: c.964G>A | p.(Gly322Arg) | enz | 1 | Mucopolysaccharidosis type IVA | AR | P |
| *GBA* | NM_000157.3: c.1053G>C | p.(Trp351Cys) | enz./biom. | 1 | Gaucher disease | AR | P |
| *GBA* | NM_000157.3: c.1406G>T | p.(Gly469Val) | enz./biom. | 1 | Gaucher disease | AR | P |
| *GBA* | NM_000157.3: c.1574G>A | p.(Gly525Asp) | enz./biom. | 1 | Gaucher disease | AR | P |
| *GBA* | NM_000157.3: c.263T>C | p.(Met88Thr) | enz./biom. | 2 | Gaucher disease | AR | P |
| *GBA* | NM_000157.3: c.352A>G | p.(Lys118Glu) | enz./biom. | 1 | Gaucher disease | AR | P |
| *GBA* | NM_000157.3: c.464A>G | p.(Tyr155Cys) | enz./biom. | 1 | Gaucher disease | AR | P |
| *GBA* | NM_000157.3: c.521A>C | p.(Tyr174Ser) | enz./biom. | 1 | Gaucher disease | AR | P |
| *GBA** | NM_000157.3: c.710A>C | p.(Lys237Thr) | enz./biom. | 1 | Gaucher disease | AR | P |
| *GLA* | NM_000169.2: c.683dup | p.(Asn228Lysfs*4) | enz./biom. | 1 | Fabry disease | XL | P |
| *GLB1* | NM_001317040.1: c.1026T>G | p.(Tyr342*) | enz | 1 | Mucopolysaccharidosis type IVB | AR | P |
| *GLB1* | NM_001317040.1: c.1058+5G>A |  | enz | 1 | Mucopolysaccharidosis type IVB | AR | P |
| *GLB1* | NM_001317040.1:c.1059-1G>A |  | enz | 1 | Mucopolysaccharidosis type IVB | AR | P |
| *GLB1* | NM_001317040.1: c.1107C>G | p.(Asn369Lys) | enz | 1 | Mucopolysaccharidosis type IVB | AR | P |
| *GLB1* | NM_001317040.1: c.1726G>T | p.(Gly576*) | enz | 1 | Mucopolysaccharidosis type IVB | AR | P |
| *GLB1* | NM_001317040.1: c.1850C>T | p.(Thr617Ile) | enz | 3 | Mucopolysaccharidosis type IVB | AR | P |
| *GLB1* | NM_001317040.1: c.1922del | p.(Pro641Glnfs*7) | enz | 1 | Mucopolysaccharidosis type IVB | AR | P |
| *GLB1* | NM_001317040.1: c.308T>C | p.(Ile103Thr) | enz | 1 | Mucopolysaccharidosis type IVB | AR | P |
| *GLB1* | NM_001317040.1: c.584T>G | p.(Leu195Arg) | enz | 1 | Mucopolysaccharidosis type IVB | AR | P |
| *GLB1* | NM_001317040.1: c.602-1G>C |  | enz | 1 | Mucopolysaccharidosis type IVB | AR | P |
| *GLB1* | NM_001317040.1: c.627G>T | p.(Trp209Cys) | enz | 1 | Mucopolysaccharidosis type IVB | AR | LP |
| *GLB1* | NM_001317040.1: c.629del | p.(Leu210Trpfs*8) | enz | 1 | Mucopolysaccharidosis type IVB | AR | P |
| *GLB1* | NM_001317040.1: c.878-8A>G |  | enz | 1 | Mucopolysaccharidosis type IVB | AR | P |
| *GUSB* | NM_000181.3: c.1065+5G>A |  | enz | 2 | Mucopolysaccharidosis type VII | AR | P |
| *HEXA* | NM_001318825.1: c.1363G>A | p.(Gly455Ser) | enz | 2 | Tay-Sachs disease | AR | P |
| *HEXA* | NM_001318825.1: c.1415_1417dup | p.(Gly472dup) | enz | 1 | Tay-Sachs disease | AR | P |
| *HEXA* | NM_001318825.1: c.492+3A>T |  | enz | 1 | Tay-Sachs disease | AR | P |
| *HEXA* | NM_001318825.1: c.652G>A | p.(Asp218Asn) | enz | 1 | Tay-Sachs disease | AR | P |
| *HEXA* | NM_001318825.1: c.751A>T | p.(Lys251*) | enz | 1 | Tay-Sachs disease | AR | P |
| *HEXA* | NM_001318825.1: c.895G>A | p.(Gly299Arg) | enz | 1 | Tay-Sachs disease | AR | P |
| *HEXB* | NM_000521.3: c.1270T>G | p.(Trp424Gly) | enz | 1 | Sandhoff disease | AR | P |
| *HEXB* | NM_000521.3: c.1303_1304insCT | p.(Arg435Thrfs*10) | enz | 1 | Sandhoff disease | AR | P |
| *HEXB* | NM_000521.3: c.1417+5G>A |  | enz | 2 | Sandhoff disease | AR | P |
| *HEXB* | NM_000521.3: c.325dup | p.(Tyr109Leufs*6) | enz | 1 | Sandhoff disease | AR | LP |
| *HEXB* | NM_000521.3: c.535_536del | p.(Val179Leufs*12) | enz | 1 | Sandhoff disease | AR | P |
| *HEXB* | NM_000521.3: c.826_829del | p.(Glu276Metfs*30) | enz | 1 | Sandhoff disease | AR | P |
| *HEXB* | NM_000521.3: c.892del | p.(Trp298Glyfs*9) | enz | 2 | Sandhoff disease | AR | P |
| *IDS* | NM_000202.5: c.1006+1G>A |  | enz | 1 | Mucopolysaccharidosis type II | XL | P |
| *IDS* | NM_000202.5: c.1006+1G>C |  | enz | 2 | Mucopolysaccharidosis type II | XL | P |
| *IDS* | NM_000202.5: c.1007-2del |  | enz | 2 | Mucopolysaccharidosis type II | XL | P |
| *IDS* | NM_000202.5: c.103+1G>A |  | enz | 1 | Mucopolysaccharidosis type II | XL | P |
| *IDS* | NM_000202.5: c.1035G>C | p.(Trp345Cys) | enz | 1 | Mucopolysaccharidosis type II | XL | P |
| *IDS* | NM_000202.5: c.1036G>A | p.(Ala346Thr) | enz | 1 | Mucopolysaccharidosis type II | XL | LP |
| *IDS* | NM_000202.5: c.112_116del | p.(Asn38Serfs*7) | enz | 1 | Mucopolysaccharidosis type II | XL | LP |
| *IDS* | NM_000202.5: c.118del | p.(Leu40Phefs*20) | enz | 1 | Mucopolysaccharidosis type II | XL | P |
| *IDS* | NM_000202.5: c.1215del | p.(Leu406Phefs*34) | enz | 1 | Mucopolysaccharidosis type II | XL | P |
| *IDS* | NM_000202.5: c.322T>G | p.(Tyr108Asp) | enz | 1 | Mucopolysaccharidosis type II | XL | P |
| *IDS* | NM_000202.5: c.329G>A | p.(Arg110Lys) | enz | 1 | Mucopolysaccharidosis type II | XL | P |
| *IDS* | NM_000202.5: c.486_489del | p.(Glu163Serfs*49) | enz | 2 | Mucopolysaccharidosis type II | XL | LP |
| *IDS* | NM_000202.5: c.619_627delinsTTGAT | p.(Gln207Leufs*5) | enz | 1 | Mucopolysaccharidosis type II | XL | LP |
| *IDS* | NM_000202.5: c.686A>C | p.(His229Pro) | enz | 2 | Mucopolysaccharidosis type II | XL | P |
| *IDUA* | NM_000203.3: c.1045G>C | p.(Asp349His) | enz | 1 | Mucopolysaccharidosis type I | AR | P |
| *IDUA* | NM_000203.3: c.1324_1330del | p.(Ala442Thrfs*13) | enz | 1 | Mucopolysaccharidosis type I | AR | P |
| *IDUA* | NM_000203.3: c.1350del | p.(Asn451Thrfs*6) | enz | 1 | Mucopolysaccharidosis type I | AR | P |
| *IDUA* | NM_000203.3: c.1599del | p.(Ser534Argfs*26) | enz | 1 | Mucopolysaccharidosis type I | AR | P |
| *IDUA* | NM_000203.3: c.1641dup | p.(Pro548Alafs*24) | enz | 1 | Mucopolysaccharidosis type I | AR | P |
| *IDUA* | NM_000203.3: c.1726A>G | p.(Lys576Glu) | enz | 1 | Mucopolysaccharidosis type I | AR | P |
| *IDUA* | NM_000203.3: c.1875C>G | p.(Tyr625*) | enz | 1 | Mucopolysaccharidosis type I | AR | P |
| *IDUA* | NM_000203.3: c.395T>C | p.(Leu132Pro) | enz | 1 | Mucopolysaccharidosis type I | AR | P |
| *IDUA* | NM_000203.3: c.494-1G>C |  | enz | 1 | Mucopolysaccharidosis type I | AR | P |
| *IDUA* | NM_000203.3: c.568_581del | p.(Asn190Hisfs*204) | enz | 1 | Mucopolysaccharidosis type I | AR | P |
| *IDUA* | NM_000203.3: c.613T>C | p.(Cys205Arg) | enz | 1 | Mucopolysaccharidosis type I | AR | P |
| *LIPA* | NM_000235.3: c.1122T>G | p.(His374Gln) | enz | 1 | Wolman disease/Cholesteryl ester storage disease | AR | LP |
| *LIPA* | NM_000235.3: c.170A>G | p.(Asp57Gly) | enz | 1 | Wolman disease/Cholesteryl ester storage disease | AR | LP |
| *LIPA* | NM_000235.3: c.221C>T | p.(Ser74Phe) | enz | 1 | Wolman disease/Cholesteryl ester storage disease | AR | P |
| *LIPA* | NM_000235.3: c.285G>C | p.(Trp95Cys) | enz | 1 | Wolman disease/Cholesteryl ester storage disease | AR | LP |
| *LIPA* | NM_000235.3: c.421G>C | p.(Ala141Pro) | enz | 2 | Wolman disease/Cholesteryl ester storage disease | AR | P |
| *LIPA* | NM_000235.3: c.676-2A>T |  | enz | 1 | Wolman disease/Cholesteryl ester storage disease | AR | P |
| *LIPA* | NM_000235.3: c.883C>A | p.(His295Asn) | enz | 1 | Wolman disease/Cholesteryl ester storage disease | AR | P |
| *MAN2B1* | NM_000528.3: c.437-1G>A |  | enz | 1 | Alpha-mannosidosis | AR | P |
| *NAGLU* | NM_000263.3: c.1162C>T | p.(Gln388*) | enz | 1 | Mucopolysaccharidosis type IIIB | AR | P |
| *NAGLU* | NM_000263.3: c.1277G>A | p.(Gly426Asp) | enz | 1 | Mucopolysaccharidosis type IIIB | AR | P |
| *NAGLU* | NM_000263.3: c.1560_1561delinsTT | p.(Pro521Ser) | enz | 1 | Mucopolysaccharidosis type IIIB | AR | P |
| *NAGLU* | NM_000263.3: c.1592A>G | p.(Tyr531Cys) | enz | 1 | Mucopolysaccharidosis type IIIB | AR | P |
| *NAGLU* | NM_000263.3: c.179del | p.(Pro60Argfs*62) | enz | 1 | Mucopolysaccharidosis type IIIB | AR | P |
| *NAGLU* | NM_000263.3: c.1877G>A | p.(Arg626Gln) | enz | 1 | Mucopolysaccharidosis type IIIB | AR | LP |
| *NAGLU* | NM_000263.3: c.214_237dup | p.(Ala72_Gly79dup) | enz | 1 | Mucopolysaccharidosis type IIIB | AR | P |
| *NAGLU* | NM_000263.3: c.2190del | p.(Phe731Serfs*76) | enz | 1 | Mucopolysaccharidosis type IIIB | AR | P |
| *NAGLU* | NM_000263.3: c.398A>G | p.(Gln133Arg) | enz | 1 | Mucopolysaccharidosis type IIIB | AR | P |
| *NAGLU* | NM_000263.3: c.531+1G>T |  | enz | 1 | Mucopolysaccharidosis type IIIB | AR | P |
| *NPC1* | NM_000271.4: c.1180_1181dup | p.(Phe395Thrfs*55) | biom. | 1 | Niemann-Pick disease type C1 | AR | P |
| *NPC1* | NM_000271.4: c.1610T>C | p.(Phe537Ser) | biom. | 1 | Niemann-Pick disease type C1 | AR | P |
| *NPC1* | NM_000271.4: c.1757+2T>C |  | biom. | 1 | Niemann-Pick disease type C1 | AR | P |
| *NPC1* | NM_000271.4: c.2084T>C | p.(Leu695Pro) | biom. | 1 | Niemann-Pick disease type C1 | AR | P |
| *NPC1* | NM_000271.4: c.2285T>C | p.(Leu762Pro) | biom. | 1 | Niemann-Pick disease type C1 | AR | P |
| *NPC1* | NM_000271.4: c.2596A>G | p.(Met866Val) | biom. | 1 | Niemann-Pick disease type C1 | AR | LP |
| *NPC1* | NM_000271.4: c.270_273dup | p.(Gln92Serfs*13) | biom | 1 | Niemann-Pick disease type C1 | AR | P |
| *NPC1* | NM_000271.4: c.2758G>A | p.(Val920Met) | biom | 2 | Niemann-Pick disease type C1 | AR | P |
| *NPC1* | NM_000271.4: c.3041+2dup |  | biom | 1 | Niemann-Pick disease type C1 | AR | P |
| *NPC1* | NM_000271.4: c.3154_3156del | p.(Ile1052del) | biom | 1 | Niemann-Pick disease type C1 | AR | P |
| *NPC1* | NM_000271.4: c.3185C>A | p.(Ala1062Asp) | biom | 1 | Niemann-Pick disease type C1 | AR | P |
| *NPC1* | NM_000271.4: c.3314C>A | p.(Ser1105Tyr) | biom | 1 | Niemann-Pick disease type C1 | AR | P |
| *NPC1* | NM_000271.4: c.3412_3413del | p.(Met1138Valfs*119) | biom | 1 | Niemann-Pick disease type C1 | AR | P |
| *NPC1* | NM_000271.4: c.3460_3471del | p.(Leu1154_Leu1157del) | biom | 1 | Niemann-Pick disease type C1 | AR | LP |
| *NPC1* | NM_000271.4: c.3507C>G | p.(Ser1169Arg) | biom | 2 | Niemann-Pick disease type C1 | AR | P |
| *NPC1* | NM_000271.4: c.3590C>T | p.(Ser1197Phe) | biom | 2 | Niemann-Pick disease type C1 | AR | P |
| *NPC1* | NM_000271.4: c.428_429del | p.(Glu143Valfs*26) | biom | 1 | Niemann-Pick disease type C1 | AR | P |
| *NPC1* | NM_000271.4: c.433C>T | p.(Gln145*) | biom | 1 | Niemann-Pick disease type C1 | AR | P |
| *NPC1* | NM_000271.4: c.754del | p.(Gln252Serfs*58) | biom | 1 | Niemann-Pick disease type C1 | AR | P |
| *NPC1* | NM_000271.4: c.803_805dup | p.(Met268_Tyr269insLeu) | biom | 1 | Niemann-Pick disease type C1 | AR | P |
| *NPC2* | NM_006432.3: c.385C>T | p.(Gln129*) | biom | 1 | Niemann-Pick disease type C2 | AR | P |
| *SMPD1* | NM_000543.4: c.1091+1G>T |  | enz./biom. | 1 | Niemann-Pick disease type A/B | AR | P |
| *SMPD1* | NM_000543.4: c.1155T>G | p.(Asn385Lys) | enz./biom. | 1 | Niemann-Pick disease type A/B | AR | P |
| *SMPD1* | NM_000543.4: c.1178G>C | p.(Trp393Ser) | enz./biom. | 1 | Niemann-Pick disease type A/B | AR | P |
| *SMPD1* | NM_000543.4: c.132_136del | p.(Leu45Alafs*5) | enz./biom. | 1 | Niemann-Pick disease type A/B | AR | P |
| *SMPD1* | NM_000543.4: c.1345G>A | p.(Glu449Lys) | enz./biom. | 1 | Niemann-Pick disease type A/B | AR | LP |
| *SMPD1* | NM_000543.4: c.1382_1383del | p.(His461Argfs*3) | enz./biom. | 1 | Niemann-Pick disease type A/B | AR | P |
| *SMPD1* | NM_000543.4: c.1521_1531del | p.(Gly508Argfs*6) | enz./biom. | 1 | Niemann-Pick disease type A/B | AR | P |
| *SMPD1* | NM_000543.4: c.1566T>G | p.(Asn522Lys) | enz./biom. | 2 | Niemann-Pick disease type A/B | AR | P |
| *SMPD1* | NM_000543.4: c.1611del | p.(Leu538Serfs*75) | enz./biom. | 1 | Niemann-Pick disease type A/B | AR | P |
| *SMPD1* | NM_000543.4: c.1640T>C | p.(Leu547Pro) | enz./biom. | 1 | Niemann-Pick disease type A/B | AR | P |
| *SMPD1* | NM_000543.4: c.1775T>C | p.(Leu592Pro) | enz./biom. | 1 | Niemann-Pick disease type A/B | AR | P |
| *SMPD1* | NM_000543.4: c.188dup | p.(Leu64Serfs*78) | enz./biom. | 1 | Niemann-Pick disease type A/B | AR | P |
| *SMPD1* | NM_000543.4: c.421_517del | p.(Glu141Serfs*84) | enz./biom. | 1 | Niemann-Pick disease type A/B | AR | P |
| *SMPD1* | NM_000543.4: c.442T>C | p.(Trp148Arg) | enz./biom. | 1 | Niemann-Pick disease type A/B | AR | P |
| *SMPD1* | NM_000543.4: c.501T>G | p.(Cys167Trp) | enz./biom. | 1 | Niemann-Pick disease type A/B | AR | P |
| *SMPD1* | NM_000543.4: c.629A>G | p.(His210Arg) | enz./biom. | 1 | Niemann-Pick disease type A/B | AR | P |
| *SMPD1* | NM_000543.4: c.647T>C | p.(Leu216Pro) | enz./biom. | 1 | Niemann-Pick disease type A/B | AR | P |
| *SMPD1* | NM_000543.4: c.682T>G | p.(Cys228Gly) | enz./biom. | 1 | Niemann-Pick disease type A/B | AR | P |
| *SMPD1* | NM_000543.4: c.686G>A | p.(Cys229Tyr) | enz./biom. | 1 | Niemann-Pick disease type A/B | AR | P |
| *SMPD1* | NM_000543.4: c.894_902del | p.(Thr300_Thr302del) | enz./biom. | 2 | Niemann-Pick disease type A/B | AR | P |
| *SMPD1* | NM_000543.4: c.938_942delinsTGGTGGGCCAGCATGGT | p.(Pro313_Val314delinsLeuValGlyGlnHisGly) | enz./biom. | 1 | Niemann-Pick disease type A/B | AR | P |
| *SMPD1* | NM_000543.4: c.946C>T | p.(Pro316Ser) | enz./biom. | 1 | Niemann-Pick disease type A/B | AR | P |
| *SMPD1* | NM_001318088.1: c.170+5A>G |  | enz./biom. | 2 | Niemann-Pick disease type A/B | AR | P |
| *SUMF1* | NM_182760.3: c.514C>T | p.(Gln172*) | enz. | 1 | Multiple sulfatase deficiency | AR | LP |
| *TPP1* | NM_000391.3: c.1145+2T>G |  | enz. | 1 | Neuronal ceroid lipofuscinosis type 2 | AR | P |
| *TPP1* | NM_000391.3: c.1222A>C | p.(Ser408Arg) | enz. | 1 | Neuronal ceroid lipofuscinosis type 2 | AR | P |
| *TPP1* | NM_000391.3: c.1555del | p.(Thr519Profs*13) | enz. | 1 | Neuronal ceroid lipofuscinosis type 2 | AR | P |
| *TPP1* | NM_000391.3: c.442del | p.(Thr148Profs*4) | enz. | 1 | Neuronal ceroid lipofuscinosis type 2 | AR | P |
| *TPP1* | NM_000391.3: c.617G>C | p.(Arg206Pro) | enz. | 3 | Neuronal ceroid lipofuscinosis type 2 | AR | P |
| *TPP1* | NM_000391.3: c.764C>T | p.(Ala255Val) | enz. | 3 | Neuronal ceroid lipofuscinosis type 2 | AR | P |
| *GBA* | chr1:155204675-155205659  NM_000157 | Exons 9-11 (deletion) | enz./biom. | 1 | Gaucher disease | AR | P |
| *FUCA1* | chr1:24175130-24175334  NM_000147 | Exon 6 (deletion) | enz | 1 | Fucosidosis | AR | P |
| *GLB1* | chr3:33038532-33138706  NM_001317040 | Exons 1-17 (deletion) | enz | 1 | Mucopolysaccharidosis type IVB | AR | P |
| *GLB1* | chr3:33055539-33065827  NM_001317040 | Exons 12-16 (deletion) | enz | 1 | Mucopolysaccharidosis type IVB | AR | P |
| *IDUA* | chr4:996510-998191  NM_000203 | Exons 9-14 (deletion) | enz | 1 | Mucopolysaccharidosis type I | AR | LP |
| *ARSB* | chr5:78073032-78260429  NM_000046 | Exons 3 –8 (deletion) | enz | 1 | Mucopolysaccharidosis type VI | AR | P |
| *LIPA* | chr10:90974580-91007418  NM_001127605 | Exons 2 -10 (deletion) | enz | 1 | Wolman disease/Cholesteryl ester storage disease | AR | P |
| *HEXA* | chr15:72636413-72648996  NM_001318825 | Exons 2-14 (deletion) | enz | 1 | Tay-Sachs disease | AR | P |
| *GALNS* | chr16:88880840-88902268  NM_000512 | Exons 7-14 (deletion) | enz | 1 | Mucopolysaccharidosis type IVA | AR | P |
| *NAGLU* | chr17:40688282-40696261  NM_000263 | Exons 1-6 (deletion) | enz | 1 | Mucopolysaccharidosis type IIIB | AR | P |
| *IDS* | chrX:148568446-148586884  NM_000202 | Exons 1-8 (deletion) | enz | 1 | Mucopolysaccharidosis type II | XL | P |

enz: enzymatic activity study, biom: biomarker concentration determination, Ref. Seq: reference sequence, MOI: Mode of inheritance, AR: autosomal recessive, XL: X-linked. Bold font: copy number variants (CNV)

**Supplementary Table 6.** List of IMDs diagnosed in this cohort.

| Disease | Mode of inheritance | OMIM | Gene | Number of patients |
| --- | --- | --- | --- | --- |
| Tangier disease | AR | 205400 | *ABCA1* | 8 |
| Progressive familial intrahepatic cholestasis-3 | AR | 602347 | *ABCB4* | 11 |
| Dubin-Johnson syndrome | AR | 237500 | *ABCC2* | 11 |
| Adrenoleukodystrophy | XL | 300100 | *ABCD1* | 4 |
| Methylmalonic aciduria and homocystinuria of the cblJ type | AR | 614857 | *ABCD4* | 1 |
| Sitosterolemia 2 | AR | 618666 | *ABCG5* | 2 |
| Sitosterolemia 1 | AR | 210250 | *ABCG8* | 2 |
| Alpha-methylacetoacetic aciduria | AR | 203750 | *ACAT1* | 4 |
| Aspartylglucosaminuria | AR | 208400 | *AGA* | 2 |
| Glycogen storage disease III | AR | 232400 | *AGL* | 40 |
| Type I primary hyperoxaluria | AR | 259900 | *AGXT* | 3 |
| Doss hepatic porphyria | AR | 612740 | *ALAD* | 1 |
| Hereditary fructose intolerance | AR | 229600 | *ALDOB* | 17 |
| Congenital disorder of glycosylation type Id | AR | 601110 | *ALG3* | 1 |
| Hypophosphatasia | AD/AR | 146300/241500/241510 | *ALPL* | 5 |
| Hyaline fibromatosis syndrome | AR | 228600 | *ANTXR2* | 1 |
| Hypobetalipoproteinemia/familial hypercholesterolemia-2 | AR/AD | 615558/144010 | *APOB* | 12 |
| Apolipoprotein C-II deficiency | AR | 618677 | *APOC2* | 1 |
| Argininemia | AR | 207800 | *ARG1* | 1 |
| Metachromatic leukodystrophy | AR | 250100 | *ARSA* | 18 |
| Mucopolysaccharidosis type VI | AR | 253200 | *ARSB* | 26 |
| Farber lipogranulomatosis | AR | 228000 | *ASAH1* | 4 |
| Argininosuccinic aciduria | AR | 207900 | *ASL* | 1 |
| Classic citrullinemia | AR | 215700 | *ASS1* | 2 |
| ATP7A-related copper transport disorders | AR | 309400/304150/300489 | *ATP7A* | 4 |
| Wilson disease | **AR** | **227900** | ***ATP7B*** | **58** |
| Maple syrup urine disease | AR | 248600 | *BCKDHA* | 4 |
| Maple syrup urine disease | AR | 248600 | *BCKDHB* | 2 |
| Biotinidase deficiency | AR | 253260 | *BTD* | 16 |
| Homocystinuria with or without response to pyridoxine | AR | 236200 | *CBS* | 2 |
| Hyperalphalipoproteinemia | AD | 143470 | *CETP* | 4 |
| Neuronal ceroid lipofuscinosis-3 | AR | 204200 | *CLN3* | 4 |
| Neuronal ceroid lipofuscinosis-5 | AR | 256731 | *CLN5* | 9 |
| Neuronal ceroid lipofuscinosis-6 | AR | 601780/204300 | *CLN6* | 5 |
| Northern epilepsy | AR | 610003 | *CLN8* | 4 |
| Hereditary coproporphyria | AD/AR | 121300 | *CPOX* | 1 |
| Carbamoyl phosphate syntetase I deficiency | AR | 237300 | *CPS1* | 4 |
| Carnitine palmitoyltransferase deficiency I | AR | 255120 | *CPT1A* | 3 |
| Cystinosis | AR | 219800/219900 | *CTNS* | 6 |
| Aromatase deficiency | AR | 613546 | *CYP19A1* | 2 |
| Congenital adrenal hyperplasia | AR | 201910 | *CYP21A2* | 17 |
| Maple syrup urine disease | AR | 248600 | *DBT* | 4 |
| Dihydropyrimidine dehydrogenase | AR | 274270 | *DPYD* | 2 |
| Autosomal recessive hypophosphatemic rickets-2 | AR | 613312 | *ENPP1* | 3 |
| Familial hypercholesterolemia | AD/AR | 143890 | *EPHX2* | 1 |
| Tyrosinemia type I | AR | 276700 | *FAH* | 16 |
| Fructose-1,6-bisphosphatase deficiency | AR | 229700 | *FBP1* | 11 |
| Erythropoietic protoporphyria-1 | AR | 177000 | *FECH* | 1 |
| Fucosidosis | AR | 230000 | *FUCA1* | 1 |
| Glycogen storage disease Ia | AR | 232200 | *G6PC* | 5 |
| Nonspherocytic hemolytic anemia | XLD | 300908 | *G6PD* | 72 |
| Glycogen storage disease II | AR | 232300 | *GAA* | 18 |
| Krabbe disease | AR | 245200 | *GALC* | 12 |
| Galactosemia II | AR | 230200 | *GALK1* | 1 |
| Mucopolysaccharidosis type IVA | AR | 253000 | *GALNS* | 50 |
| Classic galactosemia | AR | 230400 | *GALT* | 18 |
| Gaucher disease | **AR** | **230800/608013** | ***GBA*** | **145** |
| Fabry disease | XL | 301500 | *GLA* | 12 |
| GLB1-related disorders | AR | 230500 /230600/230650/253010 | *GLB1* | 38 |
| GNPTAB-related disorders | AR | 252500/252600 | *GNPTAB* | 14 |
| Mucolipidosis III gamma | AR | 252605 | *GNPTG* | 3 |
| Mucopolysaccharidosis type VII | AR | 253220 | *GUSB* | 2 |
| Liver glycogen storage disease-0 | AR | 240600 | *GYS2* | 1 |
| Methylmalonic acidemia and homocysteinemia of the cblX type | AR | 309541 | *HCFC1* | 1 |
| Tay-Sachs disease | AR | 272800 | *HEXA* | 15 |
| Sandhoff disease | AR | 268800 | *HEXB* | 10 |
| Hemochromatosis | AR | 235200 | *HFE* | 5 |
| Alkaptonuria | AR | 203500 | *HGD* | 5 |
| Sanfilippo syndrome C | AR | 252930 | *HGSNAT* | 12 |
| Acute intermittent porphyria | AD | 176000 | *HMBS* | 4 |
| HPRT1 disorders | XL | 300322/300323 | *HPRT1* | 2 |
| Congenital adrenal hyperplasia | AR | 201810 | *HSD3B2* | 3 |
| Mucopolysaccharidosis type II | XLR | 300823 | *IDS* | 41 |
| Mucopolysaccharidosis type I | **AR** | **607014/607015/607016** | ***IDUA*** | **81** |
| Isovaleric acidemia | AR | 243500 | *IVD* | 3 |
| Danon disease | AR | 300257 | *LAMP2* | 1 |
| Familial hypercholesterolemia-1 | AD | 143890 | *LDLR* | 41 |
| Lysosomal acid lipase deficiency | AR | 278000 | *LIPA* | 11 |
| Congenital LPA deficiency | AD | 618807 | *LPA* | 1 |
| Familial lipoprotein lipase deficiency | AR | 238600 | *LPL* | 8 |
| Alpha-mannosidosis | AR | 248500 | *MAN2B1* | 4 |
| Neuronal ceroid lipofuscinosis-7 | AR | 610951 | *MFSD8* | 7 |
| Methylmalonic aciduria of the cblA complementation type | AR | 251100 | *MMAA* | 4 |
| Methylmalonic aciduria of the cblB complementation type | AR | 251110 | *MMAB* | 1 |
| Combined methylmalonic aciduria and homocystinuria type cblC | AR | 277400 | *MMACHC* | 5 |
| Combined methylmalonic aciduria and homocystinuria type cblC | AR | 277400 | *MMACHC* | 5 |
| Methylmalonic aciduria of the complementation group 'mut' | AR | 251000 | *MMUT* | 7 |
| Sanfilippo syndrome B | AR | 252920 | *NAGLU* | 26 |
| Sialidosis types I and II | AR | 256550 | *NEU1* | 8 |
| Niemann-Pick disease type C1 and type D | AR | 257220 | *NPC1* | 46 |
| Niemann-Pick disease type C2 | AR | 607625 | *NPC2* | 2 |
| Ornithine transcarbamylase deficiency | XL | 311250 | *OTC* | 7 |
| Phenylketonuria | **AR** | **261600** | ***PAH*** | **58** |
| Peroxisome biogenesis disorder 1A and 1B | AR | 214100/601539 | *PEX1* | 1 |
| Peroxisome biogenesis disorder 6A and 6B | AR | 614870/614871 | *PEX10* | 1 |
| Peroxisome biogenesis disorder 3A and 3B | AR | 614859/266510 | *PEX12* | 5 |
| Peroxisome biogenesis disorder 5A and 5B | AR | 614866/614867 | *PEX2* | 1 |
| Peroxisome biogenesis disorder 2A and 2B | AR | 214110/202370 | *PEX5* | 1 |
| Peroxisome biogenesis disorder 9B/Rhizomelic chondrodysplasia punctata type 1 | AR | 614879/215100 | *PEX7* | 1 |
| Phosphoglycerate kinase-1 deficiency | XLR | 300653 | *PGK1* | 1 |
| Glycogen storage disease type IXa | XLR | 306000 | *PHKA2* | 9 |
| Glycogen storage disease type Ixb | AR | 261750 | *PHKB* | 8 |
| Glycogen storage disease Ixc | AR | 613027 | *PHKG2* | 9 |
| Pyruvate kinase deficiency | AR | 266200 | *PKLR* | 2 |
| Variegate porphyria | AD | 176200 | *PPOX* | 1 |
| Neuronal ceroid lipofuscinosis-1 | AR | 256730 | *PPT1* | 3 |
| Atypical Gaucher disease | AR | 610539 | *PSAP* | 2 |
| Glycogen storage disease VI | AR | 232700 | *PYGL* | 10 |
| Mucopolysaccharidosis type IIIA | AR | 252900 | *SGSH* | 24 |
| Glucose transporter type 1 deficiency syndrome | AR | 606777/612126 | *SLC2A1* | 1 |
| Fanconi-Bickel syndrome | AR | 227810 | *SLC2A2* | 1 |
| Glycogen storage disease Ib, Ic | AR | 232220/232240 | *SLC37A4* | 3 |
| Cystinuria | AD/AR | 220100 | *SLC3A1* | 3 |
| Lysinuric protein intolerance | AR | 222700 | *SLC7A7* | 1 |
| Cystinuria | AD/AR | 220100 | *SLC7A9* | 3 |
| Rotor type hyperbilirubinemia | DR | 237450 | *SLCO1B1* | 1 |
| Niemann-Pick disease type A/B | **AR** | **257200/607616** | ***SMPD1*** | **86** |
| Multiple sulfatase deficiency | AR | 272200 | *SUMF1* | 3 |
| Neuronal ceroid lipofuscinosis-2 | AR | 204500 | *TPP1* | 17 |
| Crigler-Najjar syndrome type I | AR | 218800 | *UGT1A1* | 10 |
| Congenital erythropoietic porphyria | AR | 263700 | *UROS* | 1 |
